# Association Between Insulin Resistance and Luminal B Subtype Breast Cancer in Postmenopausal Women

**DOI:** 10.1097/MD.0000000000002825

**Published:** 2016-03-07

**Authors:** Sanggeun Nam, Seho Park, Hyung Seok Park, Sanghwa Kim, Jee Ye Kim, Seung Il Kim

**Affiliations:** From the Department of Surgery, Yonsei University College of Medicine, Seoul, Korea.

## Abstract

Currently, there is limited information on the clinical characteristics of breast cancer patients with insulin resistance. Hence, the purpose of this study was to investigate the association between insulin resistance and clinicopathological factors in newly diagnosed breast cancer patients without diabetes.

We assessed 760 patients with breast cancer treated between 2012 and 2014. We compared the clinicopathological characteristics between patients with and without insulin resistance using univariate and multivariate analyses, including after stratification by menopausal status. Insulin resistance was defined according to the homeostatic model assessment of insulin resistance.

Of 760 patients, 26.4% had insulin resistance. Age, menopausal status, body mass index, tumor size, histologic grade, Ki-67 expression, and breast cancer subtype significantly differed according to the presence of insulin resistance. Multivariate analysis revealed that postmenopausal status and obesity were significantly associated with insulin resistance. In postmenopausal women, older age, obesity, larger tumor size, advanced stage, and high proliferative luminal B subtype were significantly associated with insulin resistance. In contrast, in premenopausal patients, only obesity was related to insulin resistance. Multivariate analysis indicated that insulin resistance was independently correlated with obesity, larger tumor size, and the luminal B/human epidermal growth factor receptor-2-negative subtype in postmenopausal but not premenopausal patients.

Insulin resistance was significantly associated with larger tumors and proliferative luminal B subtype breast cancer in postmenopausal women only. These findings suggest that insulin resistance could mechanistically induce tumor progression and might be a good prognostic factor, and that it could represent a therapeutic target in postmenopausal patients with breast cancer.

## INTRODUCTION

Metabolic syndrome is a major health challenge of the 21st century. In the United States, the prevalence of metabolic syndrome has been reported as 33% in the adult population.^1^ Similarly, 31.3% of the Korean general population reportedly have metabolic syndrome, and the incidence rate is increasing yearly.^2^ Insulin resistance (IR) plays a central role in the pathophysiology of metabolic syndrome,^3^ which is characterized as a pathological condition where cells fail to respond to insulin.^4^

Previous studies have investigated the association between metabolic syndrome and malignancy. Metabolic syndrome is a risk factor for several cancers such as breast, colon, and endometrial cancers.^5–8^ The mechanisms underlying these associations are uncertain, but several studies have shown that IR causes chronic sustained hyperinsulinemia, which presumably plays a role in carcinogenesis.^9,10^ Some epidemiological studies have indicated that IR could be a risk factor for the development of breast cancer,^11,12^ and these findings were more significant in postmenopausal women.^7,13^ Additionally, women with IR tend to develop more proliferative cancers and present with a worse prognosis.^14^ However, despite these hypotheses, there are limited data concerning the clinicopathological characteristics of breast cancer patients with IR, particularly in Asian women.

The purpose of this study was to investigate the prevalence of IR in breast cancer patients and to examine any associations between IR and clinicopathological factors in newly diagnosed breast cancer patients without diabetes. We also explored the relationship of IR with prognostic factors according to menopausal status. To our knowledge, this is the first report to explore these relationships in Asian patients with breast cancer.

## MATERIALS AND METHODS

### Study Population

**FIGURE 1:**
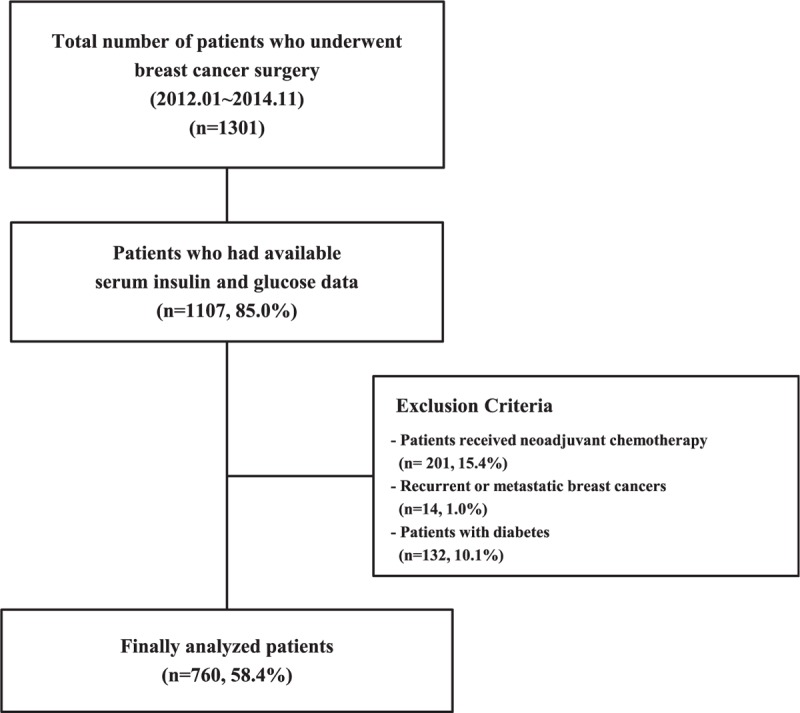
Flow diagram of patient selection.

The medical records of 1301 patients who underwent definitive surgery for breast cancer at the Department of Surgery, Yonsei University Severance Hospital in Seoul, Korea, between January 2012 and November 2014 were reviewed. Of these, 1107 had available serum insulin and glucose data. A total of 215 patients who underwent neoadjuvant chemotherapy or who were diagnosed with recurrent or metastatic breast cancers at the time of surgery were excluded. Further, 132 patients with diabetes were excluded to reduce confounding factors due to metabolic problems. Diabetes was defined as a fasting plasma glucose level ≥126 mg/dL^15^ or was determined by a previous diagnosis by a physician. The final analysis set included 760 patients (Figure 1).

This study was approved by the Institutional Review Board of Yonsei University Severance Hospital (approval number: 4-2015-0432) and was conducted in accordance with the principles described in the Declaration of Helsinki. Because of the retrospective nature of this study, the institutional review board waived the need for informed consent.

### Assessment of Insulin Resistance

Venous blood samples were taken after ≥8 hours overnight fasting to measure fasting serum glucose and insulin levels. The glucose hexokinase method was used for determining fasting blood glucose levels. The serum insulin level was measured using an electrochemiluminescence method. The presence of IR was defined using the homeostatic model assessment of insulin resistance (HOMA-IR) according to a previous study.^16^ HOMA-IR levels were calculated as follows:

HOMA-IR = fasting plasma insulin level (μIU/mL) × fasting plasma glucose level (mg/dL)/405.

The cut-off score to define IR was HOMA-IR ≥2.0, which correlated with values ≥75th percentile in our study cohort.^17,18^

### Clinicopathological Parameters

The body mass index (BMI) was defined as the weight (kg) divided by height (m) squared. According to the World Health Organization definitions for the Asian population, the subjects were categorized as normal (BMI, 18.5–24.99 kg/m^2^) or obese (BMI ≥25 kg/m^2^).^19^ Postmenopause was defined as the absence of menstruation for ≥12 months or as a serum follicle stimulating hormone level >40 mIU/mL at the time of diagnosis.

The tumor, node, metastasis (TNM) stage was established according to the criteria of the 7th American Joint Committee on Cancer Staging manual.^20^ Histologic grading followed the Nottingham grading system.^21^ High-grade ductal carcinoma in situ (DCIS) was considered as grade III disease, and non-high grade DCIS was categorized as grade I–II disease.^22^

The expressions of estrogen receptor (ER), progesterone receptor (PR), and human epidermal growth factor receptor-2 (HER-2) were immunohistochemically evaluated in formalin-fixed, paraffin-embedded whole sections of surgically resected primary breast cancer specimens. Tumors with ≥1% nuclear-stained cells were considered ER-positive or PR-positive.^23^ HER-2 staining was scored from 0 to 3+ according to the American Society of Clinical Oncology guidelines.^24^ Strong (3+) membranous staining was defined as HER-2-positive, whereas cases with 0 to 1+ were regarded as HER-2-negative. Additional fluorescence or silver in-situ hybridization was conducted in cases of equivocal (2+) staining, and an HER-2 gene-to-chromosome ratio of ≥2.0 was designated as HER-2 amplification.^25^ The breast cancer subtypes were categorized according to the St. Gallen International Expert Consensus.^26^ The Ki-67 levels were immunohistochemically measured in core-needle biopsy or surgical specimens using a primary MIB-1 antibody (Dako Denmark A/S, Glostrup, Denmark) and protocols established at the Department of Pathology at our institution. Using a visual grading system, the Ki-67 index was scored in the area with the strongest staining by counting the number of positively stained nuclei and was expressed as a percentage of total tumor cells. The Ki-67 expression was measured in core-needle biopsy specimens in 586 cases (80%) and in surgical specimens in 146 cases (20%). Tumors with ≥14% nuclear-stained cells were defined as Ki-67-positive.^27^ In cases with unavailable Ki-67 data (n = 28), the histologic grade was used to determine the breast cancer subtype, and grade III was considered as the high proliferative breast cancer subtype.^28^

### Statistical Analysis

We compared the mean serum insulin, glucose, and HOMA-IR values using independent *t* tests or 1-way analysis of variance (ANOVA) for continuous variables. The differences between categorical variables were evaluated using the chi-squared test; Fisher's exact test was used if necessary. For IR, a multivariate logistic regression model was used to calculate the odds ratio (OR) and 95% confidence interval (CI). All analyses were conducted using SPSS software version 20.0 (IBM Inc., Armonk, NY, and statistical significance was defined as a 2-tailed *P* value < 0.05.

## RESULTS

The mean age at diagnosis was 51.1 ± 10.4 years. The mean BMI was 23.0 ± 3.1 kg/m^2^, and IR was detected in 26.4% of the patients. The mean insulin, glucose, and HOMA-IR levels were 6.94 ± 5.21 μIU/mL, 96.32 ± 9.08 mg/dL, and 1.69 ± 1.38, respectively. The breast cancer stage was 0 to 1 in 67.3% of patients, and 24.4% were obese. The ER, PR, HER-2, and Ki-67 positivity rates were 76.5%, 56.5%, 20.6%, and 37.0%, respectively. According to the subtype, luminal type breast cancer accounted for 76.7% of patients, and triple-negative cancer accounted for 13.5%.

The mean insulin, glucose, and HOMA-IR levels according to clinicopathological characteristics are shown in Table 1. The mean insulin, glucose, and HOMA-IR levels were significantly higher in patients aged >50 years, postmenopausal patients, obese patients, and in those with a higher TNM stage. The mean insulin and HOMA-IR levels were significantly different according to the Ki-67 expression status and breast cancer subtype. There were no significant differences in the serum levels according to the histologic grade, ER status, or HER-2 status.

**TABLE 1:**
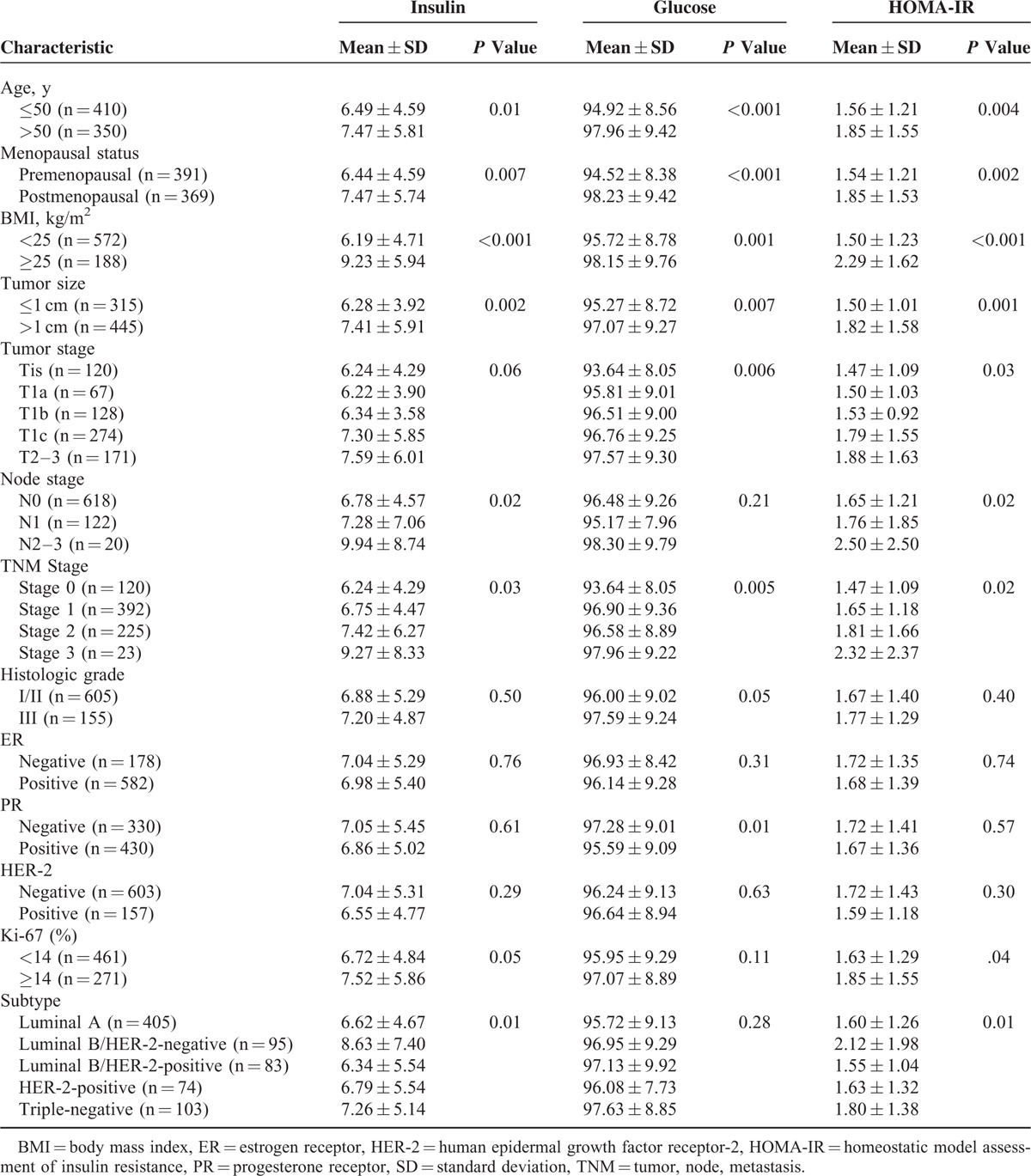
Mean Insulin, Glucose, and Homeostatic Model Assessment of Insulin Resistance (HOMA-IR) According to Clinicopathological Factors

Based on categorization by the HOMA-IR level, similar patterns between clinicopathological characteristics and IR were demonstrated (Table 2). Compared to those without IR, patients with IR tended to be older (>50 years), postmenopausal, and obese. In particular, obese patients were 2.35 times more likely to exhibit IR. Additionally, those with a tumor size >1 cm, high histologic grade, high Ki-76 index, and luminal B/HER-2-negative subtype were more likely to exhibit IR. Hormone receptor expression and HER-2 status were not associated with IR, and the TNM stage lost statistical significance when an arbitrary categorization of IR was applied. Interestingly, there was no association of individualized ER, PR, or HER-2 with IR; however, as a combined parameter, the breast cancer subtype showed a significant association with IR.

**TABLE 2:**
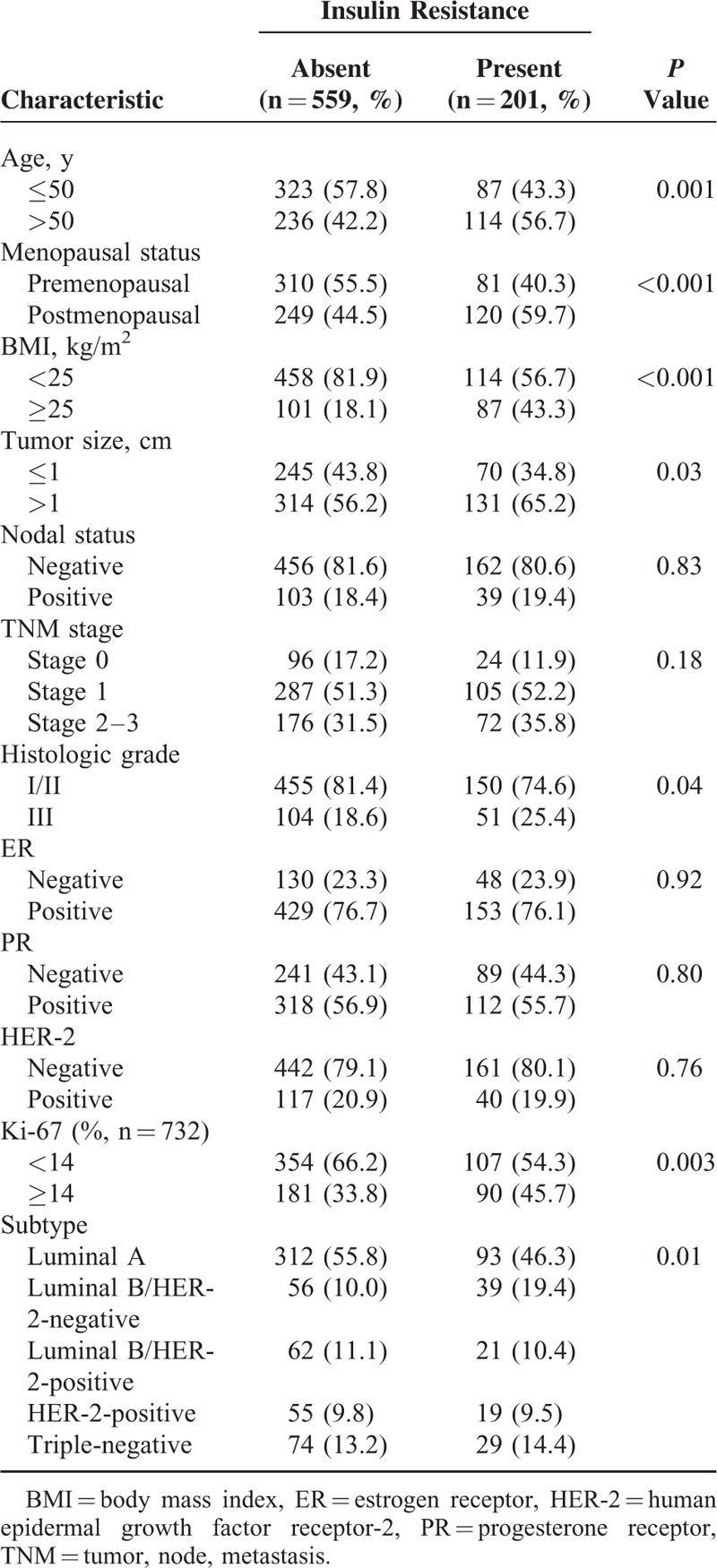
Relationships Between Insulin Resistance and Clinicopathological Factors

Clinicopathological parameters independently associated with IR according to the multivariate analyses are shown in Table 3. IR significantly correlated with postmenopausal status (OR, 1.72; 95% CI, 1.21–2.43), obesity (OR, 3.04; 95% CI, 2.12–4.37), and luminal B/HER-2-negative subtype (OR, 1.88; 95% CI, 1.13–3.14). When individualized breast cancer subtype factors such as ER, PR, HER-2, and Ki-67 status were used for the analysis instead of subtype, the postmenopausal status, obesity, and positive Ki-67 expression were significantly associated with IR.

**TABLE 3:**
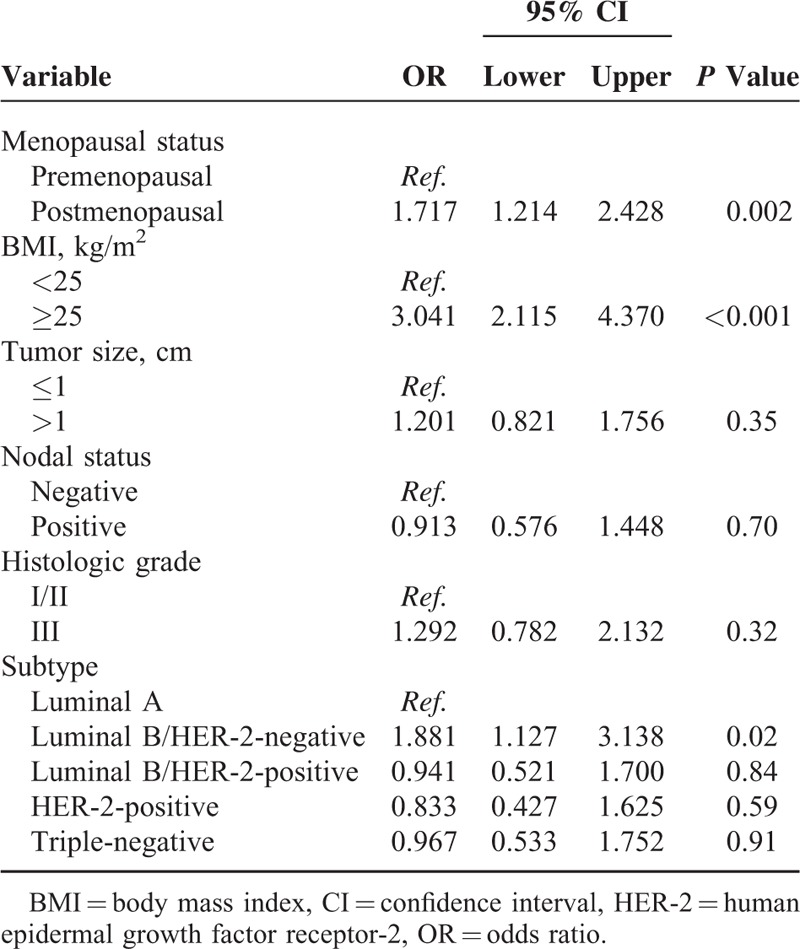
Multivariate Logistic Regression Analysis of Factors Related to Insulin Resistance

The analyses of factors associated with IR, based on stratification by menopause, are shown in Table 4. In postmenopausal women, age, BMI, tumor size, TNM stage, HER-2 status, and Ki-67 expression status were significantly different according to the presence of IR. Furthermore, in postmenopausal women, a higher proportion of patients with IR had the luminal B/HER-2-negative subtype, as compared with premenopausal women with IR. In premenopausal patients, only BMI was significantly different according to IR status.

**TABLE 4:**
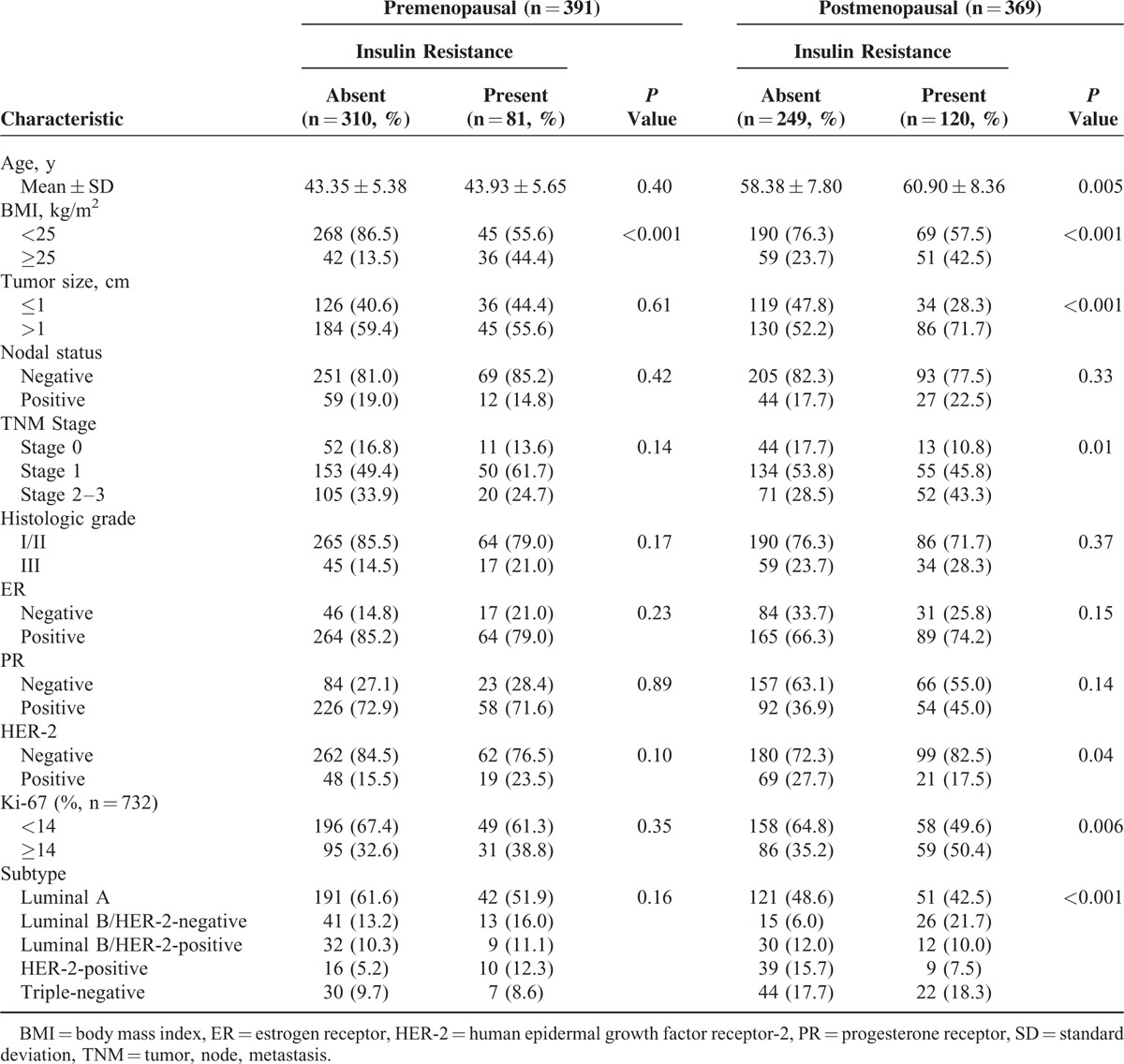
Relationships Between Insulin Resistance and Clinicopathological Factors Stratified by Menopausal Status

The multivariate analyses to identify factors related to IR in postmenopausal women are shown in Table 5. In postmenopausal patients, IR was significantly correlated with obesity (OR, 2.00; 95% CI, 1.23–3.26), higher tumor burden (OR, 1.83; 95% CI, 1.08–3.11), and luminal B/HER-2-negative subtype (OR, 2.97; 95% CI, 1.39–6.33). In premenopausal patients, only obesity was associated with IR (OR, 5.32; 95% CI, 2.99–9.50). Multivariate analysis according to subtype-related factors revealed that obesity, large tumor size, ER positivity, and Ki-67 positivity were significantly correlated with IR, and that HER-2-positivity was inversely associated with IR (data not shown).

**TABLE 5:**
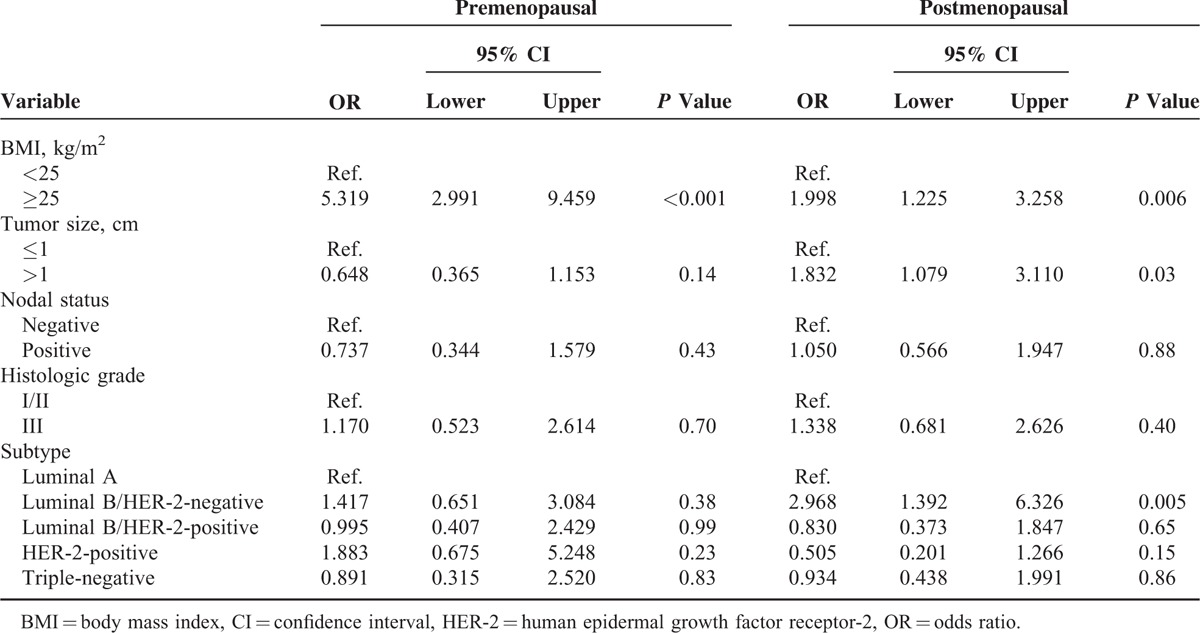
Multivariate Logistic Regression Analyses of Factors Related to Insulin Resistance by Menopausal Status

## DISCUSSION

The present study demonstrated that IR was significantly correlated with obesity, postmenopausal status, and high proliferative luminal B/HER-2-negative subtype in patients with breast cancer. Obesity is a major contributor to metabolic syndrome^3^ and is associated with high levels of circulating insulin and an increased risk of developing breast cancer, and obese patients with breast cancer are known to have a poor prognosis.^5^ Hyperinsulinemia caused by IR in obese patients is thought to contribute to carcinogenesis, although the potential mechanisms involved are unknown.^3,14^

Ki-67 is a nuclear antigen found in proliferating cells, and is therefore useful as a cellular proliferation marker.^29^ In our study, IR was positively correlated with Ki-67 expression. Insulin has diverse metabolic functions, acts as a growth factor influencing cell proliferation,^9,30^ and has a mitogenic function in normal mammary tissue, as well as in breast cancer cells.^14^ The molecular mechanisms for these associations are unknown, but several possible mechanisms have been proposed. Insulin has been found to stimulate the synthesis of insulin-like growth factor I (IGF-I) and to weakly bind to the IGF-I receptor. In turn, this insulin/IGF-I pathway could trigger signaling pathways downstream of the mitogenic-activated protein kinase and phosphoinositide-3 kinase/Akt pathways, 2 important signaling pathways contributing to carcinogenesis.^31^ These biological actions of insulin may partly explain our findings, with elevated levels of fasting insulin having been implicated in tumor progression and proliferation in breast cancer patients.^32,33^ Accordingly, our study cohort with IR harbored larger tumors and had elevated Ki-67, suggesting that IR could be a useful prognostic marker. However, the usefulness of IR as a prognostic marker requires further independent validation, including confirmation of a correlation between IR and survival in patients with breast cancer.

In the present study, IR was associated with menopausal status in patients with breast cancer. Menopause, which involves hormonal changes, can affect IR.^11^ The major endocrine change during menopause is a decrease in endogenous estradiol levels, which leads to excess androgen. This change in hormonal balance possibly contributes to an increase in visceral adiposity, which is related with IR in postmenopausal women,^34^ as observed in this study. This hormonal change can affect breast cancer pathophysiology,^35^ which is consistent with our findings that menopause was an independent risk factor for IR. Therefore, we conducted a subgroup analysis to evaluate the differences in clinicopathological characteristics according to menopausal status.

This subgroup analysis by menopausal status showed more clear associations between clinicopathological characteristics and IR among postmenopausal patients. Obesity, higher tumor burden, and the luminal B/HER-2-negative subtype were independently associated with IR in postmenopausal but not premenopausal breast cancer patients. Menopause, which involves specific hormonal changes, can affect the association between insulin resistance and breast cancer. The major source of estrogen in postmenopausal women is estrone, which is a product of the aromatization of androstenedione, formed by aromatase. This hormonal change may contribute to the increase in visceral adiposity related with IR in postmenopausal women, as observed in this study. It has been demonstrated that adipocyte-secreted factors can directly promote mammary tumorigenesis through induction of antiapoptotic transcriptional programs and proto-oncogene stabilization.^36^ These findings could facilitate the identification of subgroups that might benefit from targeted treatments aimed specifically at overcoming IR mechanisms. Prospective and randomized controlled trials to evaluate the direct antitumor effect of metformin in nondiabetic postmenopausal women with ER-positive breast cancer are ongoing,^37^ and the results of such clinical trials are highly anticipated to expand the current insights in the near future.

Unexpectedly, in our premenopausal patients, IR was not associated with traditional prognostic factors, except for BMI. This finding might suggest a differential action of IR on tumor biology in premenopausal patients with breast cancer, partly consistent with previously reported findings.^38,39^ Further investigations are required to clarify a differential metabolic action of insulin on tumor progression in premenopausal patients.

This study has several limitations. First, our findings are derived from retrospective data obtained at a single institution. Second, the gold standard to measure IR is the euglycemic glucose clamp;^40^ however, this method is impractical as it is labor- and time-intensive. Hence, we used the HOMA-IR as a surrogate for the state of IR in the present study. HOMA-IR has been observed to have a linear correlation with the euglycemic glucose clamp in various studies and is widely considered the most appropriate and extensively validated surrogate for the state of IR.^41,42^ Third, the study period was too short to assess the impact of IR as a prognostic factor in patients with breast cancer. This study relied on 1-time measurements of glucose, insulin, and HOMA-IR, which may only have a low ability to characterize a person's long-term insulin resistance along the progression and development of breast cancer. Therefore, we plan to evaluate the prognostic function of IR in the near future, using long-term retrospective data. However, despite the limitations of this study, there are also several strengths. We investigated the clinicopathological factors related to IR among newly diagnosed Asian patients with breast cancer. In particular, we examined the effects of breast cancer subtypes and Ki-67 expression for clinical relevance and found a differential association of well-known prognostic factors with IR according to menopausal status.

## CONCLUSIONS

In conclusion, IR was significantly associated with obesity, tumor proliferation, and luminal B subtype breast cancers in postmenopausal women. In premenopausal patients, IR was only associated with obesity. These findings suggest that IR might be a factor in determining prognosis in postmenopausal breast cancer and that it might facilitate the identification of subgroups that could benefit from targeted treatments to overcome IR mechanisms. Additional studies are needed to evaluate the prognosis of patients with IR and to clarify the exact role of insulin signaling pathways in patients with breast cancer, including the differential metabolic actions according to menopausal status.
